# 

**Published:** 1875-02

**Authors:** 


### CONTENTS:

### ORIGINAL COMMUNICATIONS.

ARTICLE I.—Thoroughness and Honesty in Dental Operations. By Dr. H. H. Townsend. 433
ARTICLE II.—Atrophy of the Teeth By N. M. Burkholder, D. D. S. .... 440

### SELECTED ARTICLES.

ARTICLE III.—Proceedings of American Dental Association. (Concluded.) .... 445

ARTICLE IV.—Diseases of the Enamel. By Henry S. Chase, M. D. .... 464

ARTICLE V.—Extensive Destruction of the Soft Parts near Angle of the Mouth from Sloughing after Fever. By Robt. T. Godfrey, M. D .... 468

### EDITORIAL, ETC.

Annual Commencement of the Baltimore College of Dental Surgery .... 470
Dentes Sapientiæ .... 471
A Toothless People .... 472
Correction .... 472

### MONTHLY SUMMARY.

On the Treatment of Wounds .... 473
On Salivary Fistula .... 474
Neuralgia of the Lower Jaw and Tongue treated by Excision of the Nerve .... 474
Richardson's Tooth-edge Cutting Scissors .... 475
Disinfectants .... 476
A Simple Plan of Ventilation .... 476
Moth Preventive .... 476
Colored Inks .... 477
How to Remove Rings from the Finger .... 477
Nelaton's Method of Resuscitation from Chloroform Narcosis .... 478
External Application of Iodine .... 478
Passage of Arsenic and Antimony into the Tissues and Secretions .... 478
Weight of Men and Women .... 479
Anæsthesia by Injection of Chloral into the Veins .... 479
Eucalyptus and Tobacco .... 480
American Medical Degrees .... 480

